# BdSLW-11: Dataset of Bangladeshi sign language words for recognizing 11 daily useful BdSL words

**DOI:** 10.1016/j.dib.2022.108747

**Published:** 2022-11-13

**Authors:** Md. Monirul Islam, Md. Rasel Uddin, Most Jannatul Ferdous, Sharmin Akter, Md. Nasim Akhtar

**Affiliations:** a Department of Computer Science and Engineering, University of Information Technology and Sciences (UITS), Dhaka 1212, Bangladesh; b Department of Computer Science and Engineering, Bangladesh University of Business and Technology (BUBT), Mirpur, Dhaka-1216, Bangladesh; c Department of Computer Science and Engineering, Atish Dipankar University of Science & Technology, Dhaka 1230, Bangladesh; d Department of Computer Science and Engineering, Dhaka University of Engineering & Technology, Gazipur 1707, Bangladesh

**Keywords:** Deaf & dumb community, Bangla sign language words, Image classification, Computer vision, Image processing

## Abstract

The dataset of Bangladeshi sign language words (BdSLW) is rare. Though there are lots of datasets of BdSL sign alphabets, numbers, or characters, there are not enough datasets of sign words. This is the first dataset about sign words of BdSL according to the author(s) knowledge. So, this dataset is developed by collecting data from people. This is an image dataset. This dataset is a collection of 1105 images of sign words. A total of 11 sign word categories are selected which are important and daily use in our life. As this is an image dataset, so the images of sign words are taken by camera from the sign users of Bangladesh. Authors have gone to the individuals of sign users and captured images from them with their permission. Then the images are analyzed and segmented into the images which have quality such as no background, clear, bright, etc. This dataset is used for recognizing BdSL sign words.

**Specifications Table**

**Table:** 

Subject	Computer Vision and Pattern Recognition
Specific subject area	Recognizing Bengali Sign Language (BdSL) Words
Type of data	Image
How the data were acquired	Camera; A smart phone used to capture the images of hand gestures. The images were captured on a smartphone camera at 60 fps and 720p resolution. After capturing the images, filtering and labeling are done to the images and finally, they are resized as 224 × 224 pixels.
Data format	Raw Analyzed Filtered
Description of data collection	Total collection of 1105 images of 11 distinct categories or classes. The categories are labeled as 11 sign words. Each sign word class of the dataset has more than 77 images. The images are focused on hand gestures with a clear background and in RGB format.
Data source location	Institution: Department of Computer Science and Engineering, University of Information Technology and Sciences, Dhaka-1212, Bangladesh.
Data accessibility	Repository name: Mendeley Data Data identification number: DOI: 10.17632/523d6dxz4n.4 Direct URL to data: https://data.mendeley.com/datasets/523d6dxz4n[9]
Related research article	Islam M.M., Uddin M.R., Akhtar M.A., Alam K.M.R., Recognizing Multiclass Static Sign Language for Deaf and Dumb people of Bangladesh based on Transfer Learning Techniques, Informatics in Medicine Unlocked (2022): 101,077. https://doi.org/10.1016/j.imu.2022.101077

## Value of the Data

• This dataset is greatly useful for the implementation of both image processing and image classification.
• These data are useful because they will be used to research sign language words recognition automatically.
  The Deaf and Dumb (D&D) community will greatly benefit as these data will be used by researchers to develop automatic sign language words recognition.
• The researcher of Computer Vision can use these data for further experiments to achieve better performance in recognizing sign language words.
• The dataset is essential for recognizing Bangladeshi Sign Language (BdSL) Words.
• BdSLW-11 will greatly contribute to removing Deaf and Dumb (D&D) people's communication barrier.

## 1 Objective

The main objective behind the generation of the BdSLW-11 dataset is to contribute to researchers developing a system to remove the communication barrier of the Deaf and Dumb (D&D) community of Bangladesh. This dataset will help researchers greatly to do their research in Bangladeshi Sign Language (BdSL) especially sign words. We have developed this dataset. This dataset supports an original research article that has been published. So, this data article adds value to the published research article by demonstrating the dataset functionality clearly and its effectiveness in recognizing BdSL sign words. This article will comprehensibly give an idea to readers about the BdSLW-11 dataset. This will help them to easily understand the dataset and reuse the dataset if they want to do further research in BdSL sign words.

## 2 Data Description

Sign language is different from normal languages. The Deaf and Dumb (D&D) community use this language. They use various gestures to communicate among themselves and also with normal people. Sign language is dynamic or static hand gestures [2]. D&D community suffers unexplained sufferings for communication. Computer vision can greatly contribute to eradicating their suffering. Computer vision is being used for various real-life problems [3]. Researchers are developing various models or systems using computer vision to recognize hand signs and translate them into voice or text [4]. These systems or models require a dataset to experiment with. Though there are a lot of Bangladeshi Sign Alphabets, Numbers, and characters datasets available [5], [6], [7], there is no Bangladeshi Sign Words Dataset available publicly.

**Fig. 1:**
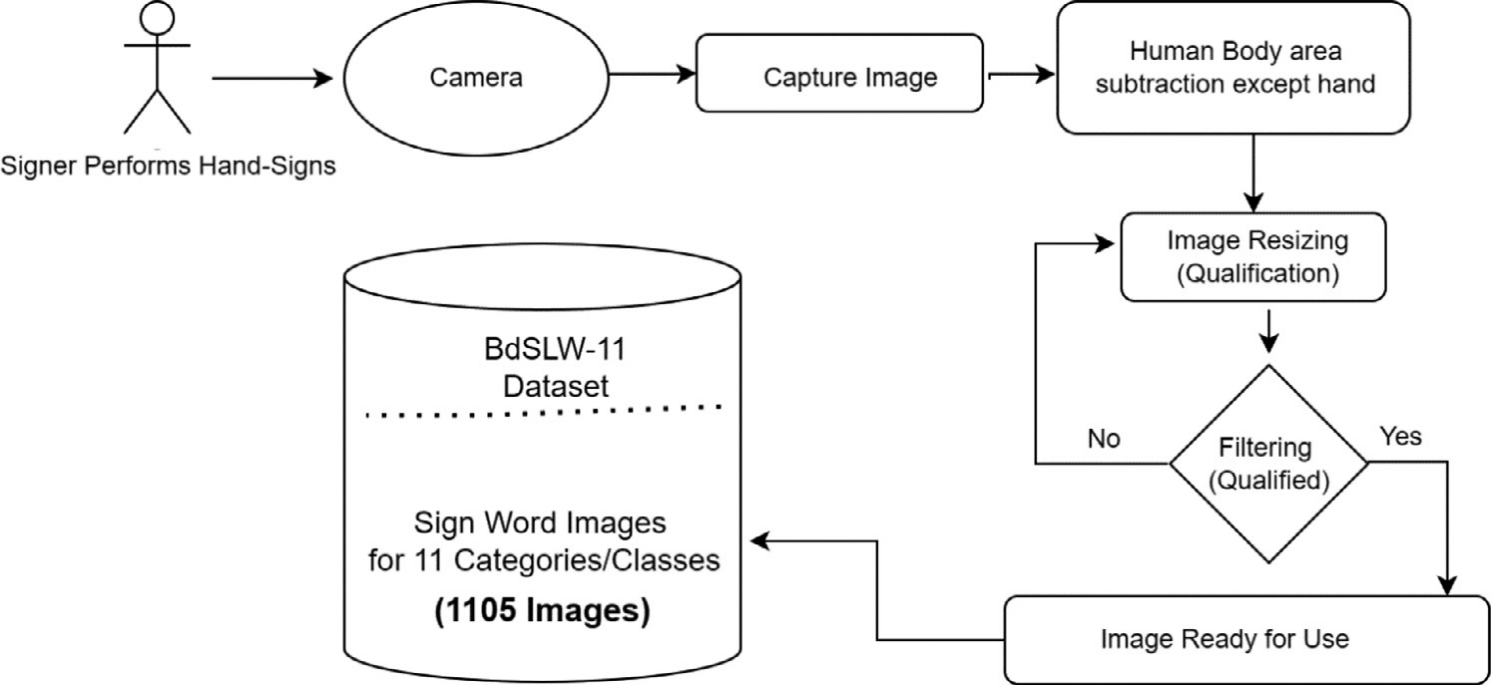
BdSLW-11 dataset creation.

**Fig. 2:**
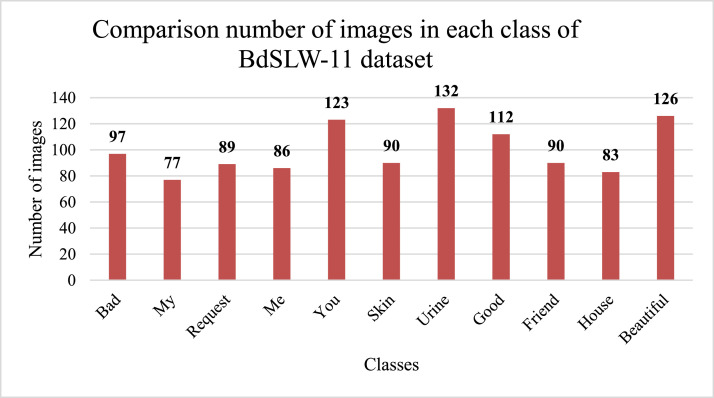
Image count of each class in the dataset.

In this work, we have developed a Bangladeshi sign language words (BdSLW) dataset for recognizing 11 daily useful common sign words. The selected sign words are 'Bad', 'Beautiful', 'Friend', 'Good', 'House', 'Me', 'My', 'Request', 'Skin', 'Urine' and 'You'. The images are processed as cropped and separated as qualified based on the focus on hand gestures, clear background, and brightness. This dataset supports an original research article [1]. Fig. 1 describes the methodology of constructing the BdSLW-11 dataset and Fig. 2 shows the total number of images in each class.

The hand sign of the 11 sign words is taken manually from real volunteer signers with their full permission. We have captured images of hung signs when the signer performs the sign. The images are then analyzed and removed from the other human body except for the hand portion. Images were resized as 224 × 224 pixels and filtered the images. If the images were qualified, they are stored in the dataset as their class. Otherwise, the images were reanalyzed until fit for use.

Fig. 2 presents the total count of images of each class/category of BdSLW-11 datasets. The dataset is a collection of sign images consisting of 1105 images. The bar chart shows that the ‘Urine’ class has the greatest number of images around 132 and the ‘My’ class contains the lowest number of images at 77. The range of total images of each class is 77 to 132. The dataset has still some limitations such as the size of the dataset. The size of the image can be increased in a future version. Besides the background of the images is kept as same for better recognition accuracy. So, there are not enough variations and lighting. Though BdSLW-11 has some limitations, this is the first Bangladeshi sign language words dataset as per author(s) knowledge.

## 3 Experimental Design, Materials and Methods

**Fig. 3:**
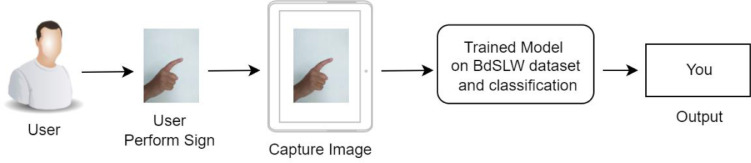
Workflow of designed method.

The proposed methods of recognizing the Bangladeshi sign language words (BdSLW) consist of two phases. One is data augmentation and the other is 11 classes of BdSLW recognition. The method is based on capturing the image of performed sign of the user and using the trained transfer learning (TL) models to classify. Finally, it displays the recognized BdSL sign words of the input sign as Fig. 3.

**Fig. 4:**
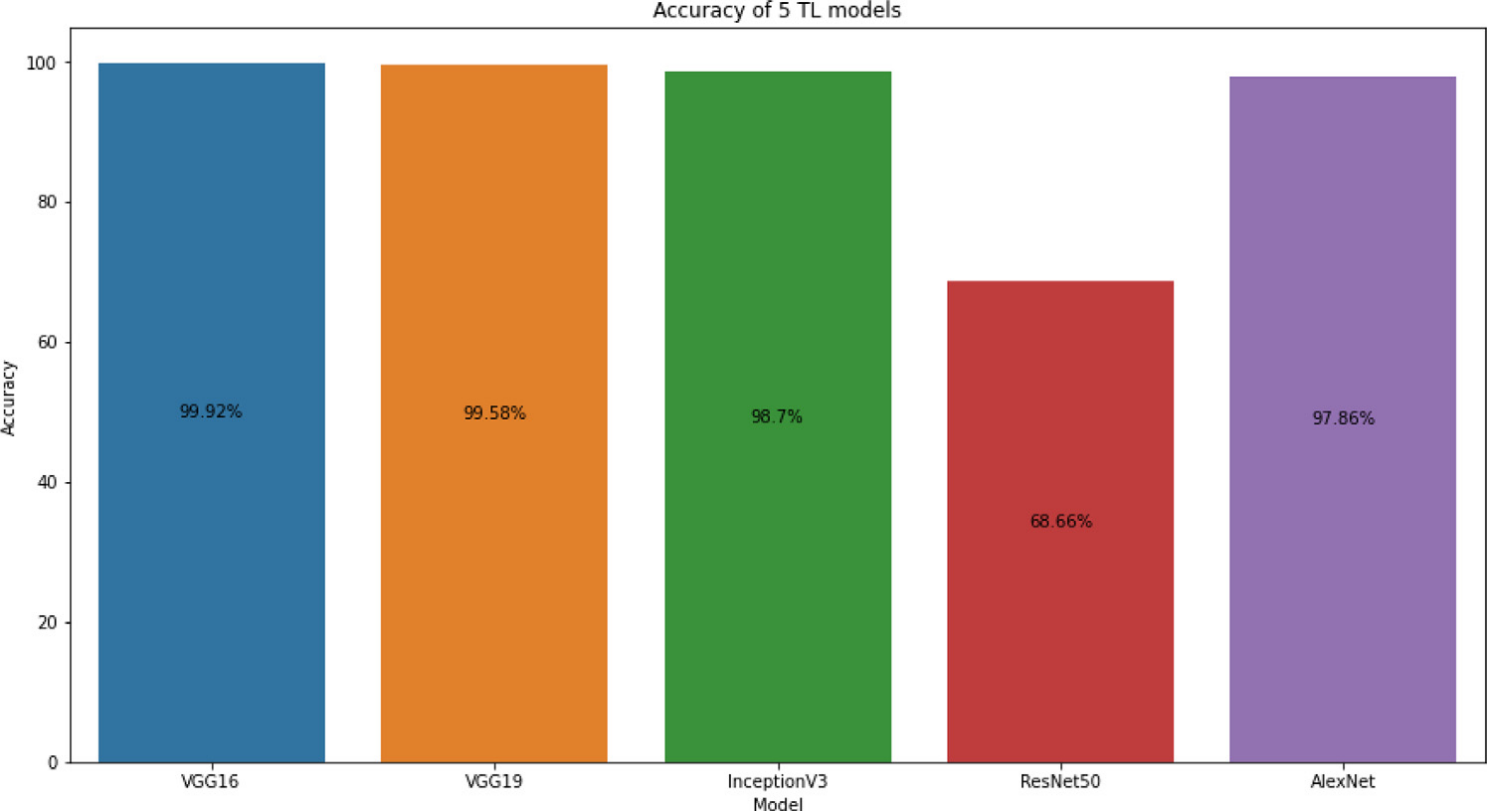
Comparison of different TL model accuracy on BdSLW-11 dataset.

The dataset is evaluated by deep learning models especially transfer learning (TL) techniques to recognize the 11 BdSL sign words. The most popular transfer learning techniques are VGG16, VGG19, InceptionV3, AlexNet, ResNet-50, Xception, and DenseNet [4]. We have used VGG16, VGG19, InceptionV3, ResNet-50, and AlexNet to train our dataset for evaluation. The accuracy of the employed TL methods that have been trained on the BdSLW-11 dataset is depicted in Fig. 4.

Fig. 4 demonstrates the accuracy of the five pre-trained models of transfer learning techniques that have been trained on our developed BdSLW-11 sign words recognition dataset. The models have achieved 99.92%, 99.58%, 98.70%, 68.66% and 97.86% accuracy for VGG16, VGG19, InceptionV3, ResNet50 and AlexNet respectively. This shows that the VGG16 model achieves the best accuracy. VGG16 TL model performs better to recognize 11 daily useful sign words comparing other models trained on our BdSLW-11 dataset.

Dataset size greatly impacts the accuracy and recognition of sign language. So before training the transfer learning techniques on our dataset, it is recommended to augment the dataset. This will enhance the size of the dataset. The implemented TL models of the above accuracy have been done on the augmented dataset. The augmented dataset size was 3835 images of the training set. We had split the 1105 images into 850 images for the training set and 255 images for the testing set. Augmentation is various techniques to enlarge the dataset such as flipping, color space, cropping, rotation, translation, noise injection, etc. [8]. We have used flipping, rotation, and zooming augmentation techniques to augment the dataset which enhances the size of the dataset. The augmentation code is as follows:

## 4 Data Augmentation

**Table:** 

DataGen=ImageDataGenerator(rotation_range=40,
width_shift_range=0.2,
height_shift_range=0.2,
shear_range=0.2,
zoom_range=0.2,
horizontal_flip=True,
fill_mode='constant',cval=125)

## Ethics Statements

As the dataset is a collection of images taken from volunteer signers, full permission was taken about their consent with written. Besides, there are also no harmful or banned device uses. A smartphone camera is used to capture hand gestures. So, there is also no impact on human bodies.

## CRediT authorship contribution statement

**Md. Monirul Islam:** Methodology, Investigation, Formal analysis, Writing – original draft, Writing – review & editing. **Md. Rasel Uddin:** Conceptualization, Methodology, Investigation, Writing – original draft, Writing – review & editing. **Most Jannatul Ferdous:** Conceptualization, Supervision, Writing – review & editing. **Sharmin Akter:** Conceptualization, Supervision, Writing – review & editing. **Md. Nasim Akhtar:** Supervision, Funding acquisition, Writing – review & editing.

## Declaration of Competing Interest

The authors declare that they have no known competing financial interests or personal relationships that could have appeared to influence the work reported in this paper.

## Data Availability

BdSLW-11: A Bangladeshi Sign Language Words Dataset for Recognizing 11 daily useful BdSL Words (Original Data) (Mendeley Data).
